# SkinViT: A transformer based method for Melanoma and Nonmelanoma classification

**DOI:** 10.1371/journal.pone.0295151

**Published:** 2023-12-27

**Authors:** Somaiya Khan, Ali Khan

**Affiliations:** 1 School of Electronics Engineering, Beijing University of Posts and Telecommunications, Beijing, China; 2 School of Computer Science and Technology, Nanjing University of Aeronautics and Astronautics, Nanjing, China; Northwestern University Feinberg School of Medicine Galter Health Sciences Library, UNITED STATES

## Abstract

Over the past few decades, skin cancer has emerged as a major global health concern. The efficacy of skin cancer treatment greatly depends upon early diagnosis and effective treatment. The automated classification of Melanoma and Nonmelanoma is quite challenging task due to presence of high visual similarities across different classes and variabilities within each class. According to the best of our knowledge, this study represents the classification of Melanoma and Nonmelanoma utilising Basal Cell Carcinoma (BCC) and Squamous Cell Carcinoma (SCC) under the Nonmelanoma class for the first time. Therefore, this research focuses on automated detection of different skin cancer types to provide assistance to the dermatologists in timely diagnosis and treatment of Melanoma and Nonmelanoma patients. Recently, artificial intelligence (AI) methods have gained popularity where Convolutional Neural Networks (CNNs) are employed to accurately classify various skin diseases. However, CNN has limitation in its ability to capture global contextual information which may lead to missing important information. In order to address this issue, this research explores the outlook attention mechanism inspired by vision outlooker, which improves important features while suppressing noisy features. The proposed SkinViT architecture integrates an outlooker block, transformer block and MLP head block to efficiently capture both fine level and global features in order to enhance the accuracy of Melanoma and Nonmelanoma classification. The proposed SkinViT method is assessed by different performance metrics such as recall, precision, classification accuracy, and F1 score. We performed extensive experiments on three datasets, Dataset1 which is extracted from ISIC2019, Dataset2 collected from various online dermatological database and Dataset3 combines both datasets. The proposed SkinViT achieved 0.9109 accuracy on Dataset1, 0.8911 accuracy on Dataset3 and 0.8611 accuracy on Dataset2. Moreover, the proposed SkinViT method outperformed other SOTA models and displayed higher accuracy compared to the previous work in the literature. The proposed method demonstrated higher performance efficiency in classification of Melanoma and Nonmelanoma dermoscopic images. This work is expected to inspire further research in implementing a system for detecting skin cancer that can assist dermatologists in timely diagnosing Melanoma and Nonmelanoma patients.

## 1 Introduction

Cancer has become a major concern in the healthcare sector, with a projected global incidence of 18.1 million in 2020 [1]. As per World Health Organization (WHO) report [2], cancer has emerged as one of the top killers worldwide, responsible for approximately 10 million fatalities in 2020 alone. Nowadays, skin cancer is one of the most common types of the disease to be detected, with an estimated 1.2 million cases reported globally in 2020. Among the two primary kinds of skin cancer, that is Melanoma and Nonmelanoma, Melanoma is more fatal than the latter. According to American Cancer Society, the estimated annual incidence rate of Melanoma in United States is about 100,000 people, with around 7,650 succumbing to it [3]. Melanoma has one of the highest incidence rates in New Zealand, with 6000 people diagnosed annually and accounting for almost 80% of all skin cancer deaths [4]. Cancer Research UK statistics show that the relative survival rate for skin cancer after 5 year is 90% [5]. The survival rate for skin cancer can be increased by early detection and treatment.

The healthcare sector has been revolutionized with the recent advancements in Artificial Intelligence (AI) [6]. The emergence of machine learning in computer vision has opened up new avenues for Computer Aided Diagnosis (CAD) [7, 8]. Over the years, CAD has made considerable progress, especially in diagnosis of cancer such as lung [9], breast [10], thyroid [11], brain [12], diabetic retinopathy [13], liver [14] etc. Due to the alarming increase in skin cancer incidence rate, there is a shortage of experienced dermatologists, which can lead to difficulties in timely skin cancer identification. Moreover, CAD tools are more efficient compared to existing clinical approaches, saving both time and cost. Therefore, a CAD system for skin cancer detection is essential.

The rapid developments and improvements in deep learning (DL) have exponentially impacted the performance of CAD systems [15]. Convolutional Neural Network (CNN), a DL algorithm, has been employed extensively in applications such as classification of images and object detection. With the advancements in CNN, DL has seen significant rise in real-world applications such as surveillance [16, 17], smart city applications [18, 19], healthcare [20] etc. The emergence of self-attention in vision based applications has paved the way for the success of transformers [21]. Vision Transformer, which employs self-attention mechanism, has displayed promising results when trained on large datasets [22].

CNN faces problems distinguishing low-level features that may result in missing crucial information. Furthermore, minimum false alarms are vital for accurate diagnosis in the medical field. We propose transformer based approach which employs outlook attention mechanism to generate fine level features for token representation that helps improve the model performance. Unlike other vision transformers that use dot product attention computation, outlook attention approach utilizes linear projection to aggregate surrounding tokens from anchor token features. This characteristic of outlook attention mechanism enhances the cost-effectiveness of the model. Furthermore, SVM with L2 kernel classifier is utilized for the classification task. The proposed SkinViT model ensures Melanoma and Nonmelanoma detection with higher accuracy. The major contributions of this research are given below:

1. Distinghuising between Melanoma and Nonmelanoma is a significant challenge owing to considerable visual interclass similarities and intraclass variations. To the best of our knowledge, this is the first work on Melanoma and Nonmelanoma classification using BCC and SCC in the Nonmelanoma class. Therefore, this work focuses on the different types of skin cancer detection with the help of CAD.
2. The datasets used for this research have imbalance class distribution which can lead to the misinterpretation of the class with the fewer image samples. Thus, we perform data augmentation technique to address the imbalance dataset issue.
3. While the visual differences in skin lesions may seem small and localized, it is crucial to consider fine level global context information for efficient recognition of the skin lesions. Therefore, we present a novel DL model named as SkinViT to efficiently integrate fine level information with outlooker and global information with transformer for more reliable classification method of Melanoma and Nonmelanoma images.
4. Furthermore, a detailed analysis of the proposed SkinViT approach with different optimizers and classifiers on three datasets to detect Melanoma and Nonmelanoma is presented and compared with other SOTA models and existing research to validate the efficacy of the SkinViT model.

The remaining sections are structured as follows: Section 2 presents the related work. Next, Section 3 describes the datasets used in this research and the proposed method. Further, Section 4 presents simulation setup and results, Section 5 provides discussion and conclusion.

## 2 Related work

Over the past few years, the massive increase in skin cancer cases has overwhelmed dermatologists. To help in the accurate differentiation and diagnosis of melanoma from other skin lesions, International Society for Digital Imaging of Skin (ISDIS) took initiative to tackle the problem of the increasing incidence rate of skin cancer by introducing an annual challenge known as International Skin Imaging Collaboration (ISIC) [23]. With all these efforts, there have been considerable interest among researchers in exploring computer vision techniques for skin cancer detection [24, 25].

In [26], Pham et al. conducted comparative analysis of different data processing methods, feature extraction methods and classifiers for melanoma classification. In their analysis, Linear Normalization as data processing, HSV as feature extraction and Balanced Random Forest classifier performed best with 74.75% accuracy on the HAM10000 dataset. In [27], Shen et al. proposed high performance data augmentation, which can be integrated to any deep learning method to classify skin lesions. Their proposed approach with efficienetb0 showed the best results with 85.3% accuracy on ISIC2018/HAM10000 dataset. In [28], Zhang et al. proposed convolutional neural network with attention residual learning (ARL-CNN) to classify skin diseases. Their proposed method achieved 91.8% AUC on the ISIC2017 dataset for binary classification task. Liu et al. [29] proposed a mid level feature representation method for learning features, and the CNN model is used as an extractor of ROI images. Their proposed method achieved 92.1% AUC in classifying melanoma and S. keratosis using the ISIC2017 dataset. In [30], Zhou et al. proposed convolutional spiking neural networks (SNN) employing spike-time-dependent plasticity (STDP) learning rate for melanoma skin lesions classification. Their proposed method showed an accuracy of 87.7% in classifying malignant melanoma and nevus skin lesions using the ISIC2018 dataset.

Gouda et al. [31] proposed pre-trained deep learning models based on transfer learning such as CNN, ResNet50, InceptionV3 and Inception ResNet for skin cancer classification. In their analysis, InceptionV3 showed the highest accuracy of 85.76% in classifying malignant and benign skin lesions using the ISIC2018 dataset. In [32], Damian et al. proposed MobileNet based model transfer learning for melanoma and nevus skin lesion classification. Their proposed method an achieved accuracy of 89.7% using the ISIC2018 dataset. In [33], Indraswari et al. proposed transfer learning technique based on the MobileNetV2 model for melanoma classification. Their proposed method showed 85% accuracy on the ISIC archive dataset. Hoang et al. [34] proposed EW-FCM+ShuffleNet based hybrid method, entropy-based weighting and first-order cumulative moment (EW-FCM) is used for segmentation and wide-ShuffleNet for classification. In their proposed method EW-FCM with wide-ShuffleNet performed best with 86.33% accuracy for multi-class classification on the HAM10000 dataset. In [35], Lopez et al. proposed CNN model based on transfer learning such as VGGNet (VGG16), for skin lesion classification. Their proposed method achieved 81.33% accuracy in classifying malignant and benign skin lesions using the ISIC2016 dataset.

In [36], Xie et al. proposed a Swin-SimAM based hybrid method for detecting melanoma where a Swin transformer is used for feature extraction and SimAM is parameter-free attention module. Their proposed method displayed 90% AUC in classifying melanoma and nonmelanoma (nevus and seborrheic keratosis). In [37], Naeem et al. introduced SCDNet approach that integrates VGG16 architecture with convolutional neural networks. Their proposed SCDNet method showed the accuracy of 96.91% on ISIC2019 dataset for multi-class skin cancer classification. Tahir et al. [38] proposed DSCC_Net which utilizes convolutional neural networks (CNN). Their proposed method demonstrated promising result with an accuracy of 94.17% on three publicly available datasets (ISIC2020, HAM10000 and DermIS) for the task of classification of multi- class skin cancer types. Table 1 details the comprehensive analysis of all the work in the literature.

**Table 1:** Comparative analysis of the related work.

Author	Method	Dataset	Classes	Accuracy	Precision	Recall	AUC
Pham et al. [26]	LN+HSV+Balanced Random Forest	HAM10000	Melanoma and Benign	74.75%	–	90.09%	–
Shen et al. [27]	Augmentation+EfficientNet	HAM10000	Multi-class	85.3%	–	78.9%	–
Zhang et al. [28]	ARL-CNN	ISIC2017	Melanoma and S. Keratosis	–	–	–	91.8%
Liu et al. [29]	Mid-level features+SVM	ISIC2017	Melanoma and S. Keratosis	–	–	–	92.1%
Zhou et al. [30]	STDP based SNN	ISIC2018/HAM10000	Melanoma and Nevus	87.7%	84.6%	90.3%	83.6%
Gouda et al. [31]	InceptionV3	ISIC2018	Malignant and Benign	85.76%	–	–	86%
Damian et al. [32]	MobileNet	ISIC2018/HAM10000	Melanoma and Nevus	89.7%	92.08%	86.41%	89.64%
Indraswari et al. [33]	MobileNetV2	ISIC Archive	Malignant and Benign	85%	83%	85%	–
Hoang et al. [34]	EW-FCM+ShuffleNet	HAM10000	Multi-class	86.33%	–	86.33%	–
Lopez et al. [35]	VGGNet (VGG16)	ISIC2016	Malignant and Benign	81.33%	79.74%	78.66%	–
Xie et al. [36]	Swin-SimAM	ISIC2017	Melanoma and Nonmelanoma (Nevus+S.Keratosis)	–	–	–	90%
Naeem et al. [37]	SCDNet(VGG16+CNN)	ISIC2019	Multi-class	96.91%	92.19%	92.18%	–
Tahir et al. [38]	DSCC_Net	ISIC2020	Multi-class	94.17%	94.28%	93.76%	99.43%

The reviewed approaches for melanoma detection showed promising results, mainly in detecting melanoma from benign dermoscopic images. The existing research predominantly focused on detecting malignant and benign lesions or melanoma and benign with either nevus or seborrheic keratosis in the benign class which are non-cancerous skin lesion types. To the best of our knowledge, no existing research considered the Melanoma and Nonmelanoma (BCC and SCC) classes which are the most common skin cancer types for the classification task. Discriminating Melanoma and Nonmelanoma is quite challenging due to high intraclass differences. Although the previous research showed promising results, but for efficient skin cancer detection, there is a further need to research Melanoma and Nonmelanoma where Nonmelanoma class has BCC and SCC. The previous work primarily focused on CNN models. CNN faces problems distinguishing low level features that may result in missing crucial information. Furthermore, minimum false alarm is vital for accurate medical diagnosis. The focus of this research is on the automatic and accurate detection of Melanoma and Nonmelanoma, which can help reduce the mortality rate due to skin cancer by early diagnosis and also help ease the burden on dermatologists. For efficient Melanoma and Nonmelanoma classification, we propose the SkinViT model based on outlooker and transformer and further aid in development of automated skin cancer detection.

## 3 Materials and methods

This section details the proposed SkinViT method and dermoscopic image datasets utilized for Melanoma and Nonmelanoma classification.

### 3.1 Dataset acquisition

This study considers the binary classification problem of Melanoma and Nonmelanoma. For this research, we considered three dermoscopic image datasets: Melanoma class and Nonmelanoma class, where Nonmelanoma comprising of Basal Cell Carcinoma and Squamous Cell Carcinoma. Dataset1 [39] is a public dataset that contains 25,331 dermoscopic images in 8 different classes. We considered only two classes, with 4521 Melanoma dermoscopic images and 3952 Nonmelanoma images. Dataset2 contains dermoscopic images collected from various online dermotological database such as DermIS [40], PH2 [41] and Dermnet-NZ [42], to get more representation of the considered classes for classification task. We considered two classes with 410 Melanoma images and 672 Nonmelanoma images. For Dataset3, we combined both datasets, Dataset1 and Dataset2, to have 4930 Melanoma images and 4624 Nonmelanoma images.

**Fig 1:**
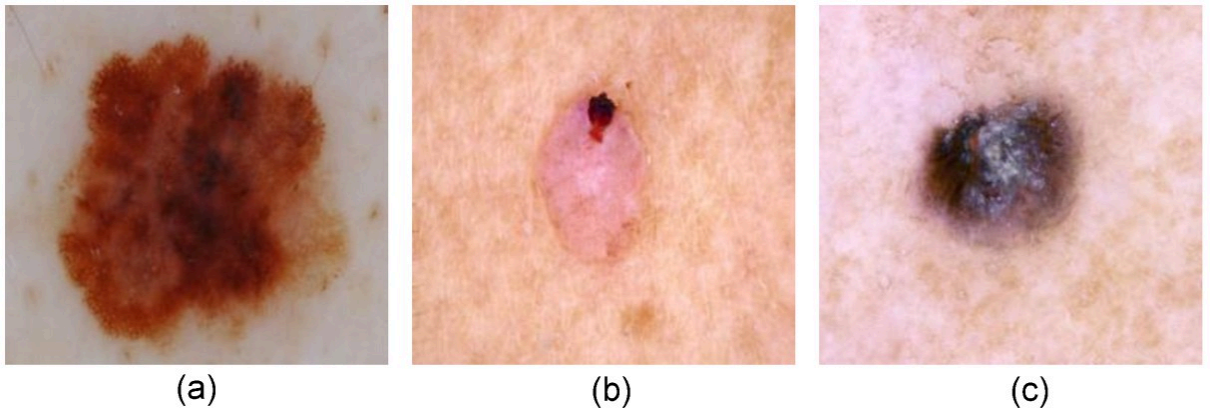
Sample Image of a) Melanoma and b) Nonmelanoma (BCC) and c) Nonmelanoma (SCC).

Melanoma is comparatively less common but the most fatal form of skin cancer. It begins in the melanocytes which is responsible for producing melanin, a pigment that gives color to the skin. It is usually a dark colored mole and changes shape, size or color over the time. Nonmelanoma is the most common kind of skin cancer and can be categorized as Basal Cell Carcinoma (BCC) and Squamous Cell Carcinoma (SCC). BCC affects the basal cell of the epidermis skin layer. BCC can have varied appearances but often appear as small pinkish or pearly-white bump. It can also be a red scaly patch sometimes with brown or black pigment within the patch. SCC is the development of keratinocytes in the squamous cell of epidermis skin layer. SCC can have variety of appearances where it typically appears as red to pink rough or scaly patch and also look like raised wart-like growth sometimes with a spiky horn-like surface sticking out. The sample images of Melanoma and Nonmelanoma are shown in Fig 1.

Fig 1(a) shows a melanoma skin lesion where it can be seen a dark brown asymmetrical shape having a diameter greater than 6mm. Fig 1(b) illustrates a BCC skin lesion having a pinkish bump with a dark brown pigment. Fig 1(c) shows an SCC skin lesion having crusty raised growth with a spiky surface sticking out.

### 3.2 Dataset preprocessing

Data preprocessing is one of the key steps in deep learning models. The dermoscopic images of Melanoma are labelled as 1, whereas the dermoscopic images of Nonmelanoma are labelled as 0. Dataset1 has a total of 8473 dermoscopic images, with 4521 images for the Melanoma class and 3952 images for the Nonmelanoma class. Dataset2 comprises 1082 dermoscopic images for the considered binary classification task, where 410 images are assigned to the Melanoma class whereas 672 images are assigned to the Nonmelanoma class. Dataset3 consists of 4930 images for Melanoma and 4624 images for the Nonmelanoma class, totaling 9554 images. The datasets are split into training and testing by applying the 80:20 splitting rule, where 80% is for training and 20% for testing purpose as depicted in Table 2. The images in datasets are of different sizes so it is essential to convert the images to a single image size to match the input of the deep learning model. Therefore, all the images in our research are converted to the image size of 224 × 224.

**Table 2:** Splitting of data.

Dataset	Classes	Training	Testing	Total
Dataset1	Melanoma	3616	905	4521
	Nonmelanoma	3162	790	3952
Dataset2	Melanoma	328	82	410
	Nonmelanoma	538	134	672
Dataset3	Melanoma	3944	986	4930
	Nonmelanoma	3700	924	4624

### 3.3 Data augmentation

The size of the data has significant impact on the performance of the deep learning models. The more the data size, the greater the chances of deep learning models to perform better. The datasets considered for our research have imbalanced data in the considered classes which can greatly impact the performance of the model. Therefore, data augmentation technique is applied to handle the imbalanced data, which can cause misinterpretation of the class with fewer sample images. For our research, we performed geometric, also known as position augmentation, on the training data, as depicted in Table 3. The images are transformed by 180° rotation, horizontal flip and shear transformation by a factor of 0.2.

**Table 3:** Data augmentations.

Augmentation	Value
Rotation	180°
Shear Transformation	0.2
Horizontal Flip	True

### 3.4 Proposed SkinViT architecture

**Fig 2:**
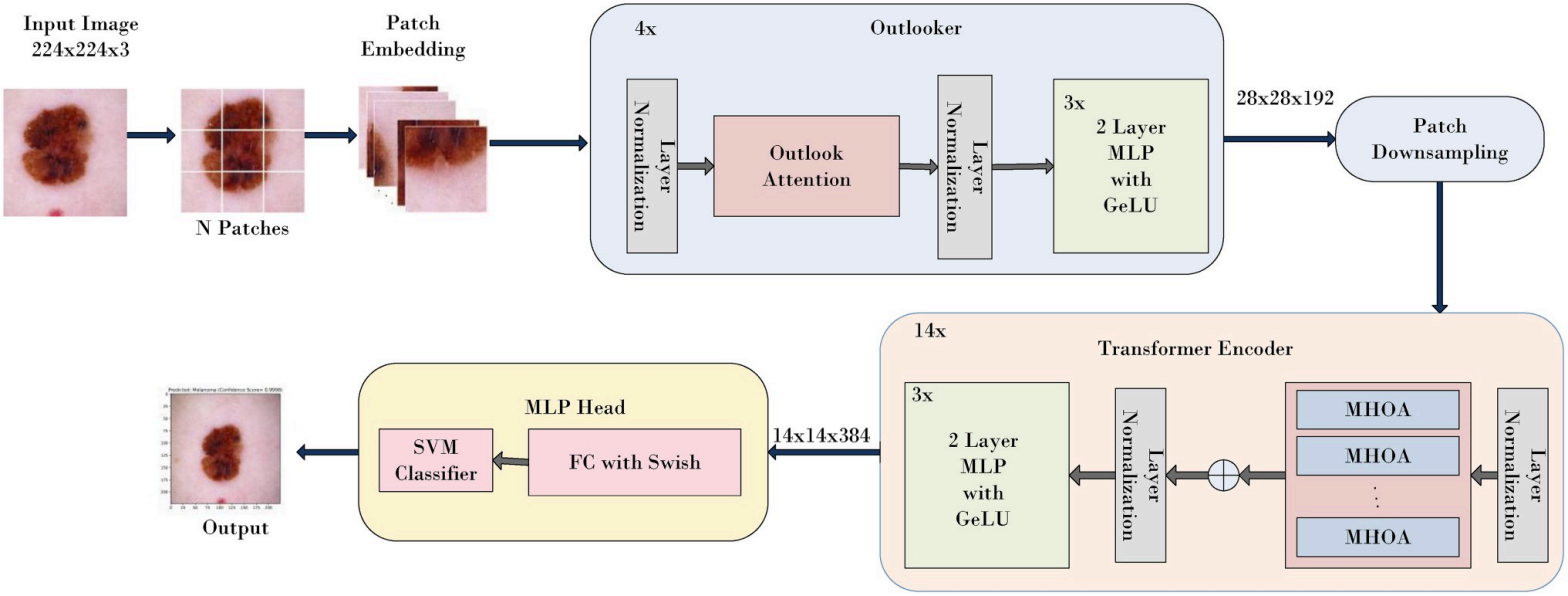
Architecture of SkinViT.

The proposed SkinViT model is designed to classify Melanoma and Nonmelanoma. The architecture of the proposed SkinViT model is depicted in Fig 2. Inspired by VOLO [43] and ViT [44], the proposed SkinViT model combines the outlooker block, transformer block and SkinViT multi-layer perceptron (MLP) head block. The proposed method first converted images into patches of size 8 × 8 which are then passed through the outlooker for generating the fine-level token representation. After that, the tokens are further down sampled using a patch embedding module which is then passed to the Transformer encoder for processing. Then the output is passed into the Multi-Layer Perceptron (MLP) head, which in our proposed SkinViT consists of flatten layer, dense layer with Swish function and a classification layer with SVM linear kernel *L*2 to output the prediction for skin cancer type. The details of the proposed SkinViT architecture is presented as follows:

1. Outlooker: The outlooker is responsible for generating fine level features for tokenization. The outlooker comprises of outlook attention layer, which encodes spatial information and MLP, which is responsible for inter-channel information interaction. The outlook attention, unlike self-attention, computes the similarity between each spatial location (*i*, *j*) and neighboring elements to focus on fine level features. For given input *X*, each *C*–dimensional feature is projected with two layers of linear weights; $A\in R^{H\times W\times K^{4}}$ as outlook weights and *V* ∈ *R*^*H*×*W*×*C*^ as value representations.
  Suppose the value representations within the local window at (*i*, *j*) are $V_{\Delta i,j}\in R^{C\times K^{2}}$ where
  $$\begin{array}{c} V_{\Delta i,j}=\{V_{i+p-\lfloor \frac{K}{2}\rfloor ,j+q-\lfloor \frac{K}{2}\rfloor }\},0\leq p,q<K \end{array}$$ (1)
  The outlook weight is reshaped into ${\hat{A}}_{\left(i,j\right)}\in R^{K^{2}\times K^{2}}$ to obtain the aggregated value of attention weight. The value projection is the weighted average of outlook weights and can be calculated as follows:
  $$\begin{array}{c} Y_{\Delta i,j}=MatMul\left(Softmax\left({\hat{A}}_{i,j}\right),V_{\Delta i,j}\right) \end{array}$$ (2)
  The outlook weight is reshaped into ${\hat{A}}_{\left(i,j\right)}\in R^{K^{2}\times K^{2}}$ to obtain the aggregated value of attention weight. The value projection is the weighted average of outlook weights and can be calculated as follows:
  $$\begin{array}{c} Y_{\Delta i,j}=MatMul\left(Softmax\left({\hat{A}}_{i,j}\right),V_{\Delta i,j}\right) \end{array}$$ (3)
  Unlike self-attention, which is dependent on query key matrix multiplications, the outlook attention matrix can be generated by attention weights within the local window located at (*i*, *j*) followed by reshape operation. Each layer of Outlooker can be written as;
  $$\begin{array}{c} \tilde{X}=OutAtt\left(LN\left(X\right)\right)+X \end{array}$$ (4)
  $$\begin{array}{c} Z=MLP\left(LN\left(\tilde{X}\right)\right)+\tilde{X} \end{array}$$ (5)
2. Transformer: The transformer encoder consists of multi-head outlook attention layers, layer normalization and MLP. The architecture of the transformer is similar to ViT, but unlike the ViT, which uses self-attention mechanism, it uses outlook attention mechanism. The multi head outlook attention is obtained by combining the computed outlook weight *A*_*n*_ and value embedding *V*_*n*_. For N number of heads, the outlook weight and value embeddings are given as $A_{n}\in R^{H\times W\times K^{4}}$ and $V_{n}\in R^{H\times W\times C_{N}}$ respectively. Here *n* = 1, 2, …..,*N* represents the dimensions of each head. The MLP in the transformer encoder in the proposed model has two layers with GeLU. Layer normalization is added before each block which helps to enhance the training performance.
3. SkinViT MLP head: The transformer encoder output is fed into the newly designed SkinViT MLP head to classify Melanoma and Nonmelanoma skin cancer. The MLP head comprises of flatten layers to flatten the output, a dense layer with Swish activation function and SVM linear kernel *L*2 as a classifier. Swish is a nonlinear and continuous function. It has a non-zero gradient for negative inputs, which allows better optimization during training. The Swish function can be written as:
  $$\begin{array}{c} f(X)=X\cdot sigmoid(X) \end{array}$$ (6)
  Where *X* is the input and *sigmoid*(*X*) is a sigmoid function that outputs the value between (0, 1).
  SVM with *L*2 Kernel: We employed linear kernel *L*2 [45] to implement SVM in our proposed method because it helps to handle the multicollinearity issue (correlated independent variables) by reducing the coefficient and maintaining all the variables. The linear kernel performs the best in the case of a large number of features. In contrast to *L*1 which uses the median of the data to estimate, linear kernel *L*2 makes a prediction based on the mean of data to prevent overfitting. *L*2 kernel includes the penalty to the cost function as the squared value of weights and learns complex patterns. *L*2 is computationally efficient and improves prediction accuracy when the output is the function of all input variables. *L*2 kernel can be calculated by:
  $$\begin{array}{c} L2reg=\lambda \sum_{i=0}^{n}w_{i}^{2} \end{array}$$ (7)
  Where *W*_*i*_ is the weight and *λ* represents the regularization parameter.
4. Optimizer: The proposed SkinViT model used Adam as an optimizer. The Adam optimizer works by computing the exponential moving average of the gradients of the parameters with respect to the loss function. It is the combination of gradient descent and momentum. The equation for the Adam optimizer is as follows:
  $$\begin{array}{c} W_{t}=W_{t-1}-\eta \frac{{\hat{M}}_{t}}{\sqrt{{\hat{V}}_{t}}+\epsilon } \end{array}$$ (8)
  Here *W* is the model weights, *η* is the step size, ${\hat{M}}_{t}$ is the unbiased estimate of the moving average of the gradient, ${\hat{V}}_{t}$ is the unbiased estimate of the moving average of the squared gradient and *ϵ* is the constant used for numerical stability having a value of 10^−8^.
5. Loss function: The binary cross entropy (*L*_*BCE*_) loss or log loss (*L*_*L*_) is often used for binary classification tasks. The *L*_*BCE*_ helps evaluate the model accuracy by determining prediction probability. The *L*_*BCE*_ computes the difference between actual probability and prediction probability and can be calculated as:
  $$\begin{array}{c} L_{BCE}=x\cdot log\left(\hat{x}\right)+\left(1-x\right)\cdot log\left(1-\hat{x}\right) \end{array}$$ (9)
  Where *x* is the label i.e. 1 for Melanoma and 0 for Nonmelanoma, and $\hat{x}$ is the predicted probability of *x*.

**Pseudocode:**

Step 1: Data Preparation

dataset = preprocess_images(labeled_images) // [*N* × *H* × *W* × *C*] array of preprocessed images

training_set, test_set = split_dataset(dataset) // [*N*_*train*, *H*, *W*, *C*], [*N*_*test*, *H*, *W*, *C*] arrays

Step 2: Model Architecture

model = create_SkinViT(num_transformer_blocks, num_outlooker_blocks, embedding_size, num_attention_heads) // SkinViT model

Step 3: Training

initialize_weights(model) // initialize model weights randomly

for epoch in 1 to num_epochs:

for batch in training_set:

accuracy = calculate_accuracy(model, batch) // calculate accuracy between model predictions and ground truth labels

update_weights(model, accuracy) // update model weights using Adam

test_accuracy = evaluate(model, test_set) // calculate test accuracy

if test_accuracy does not improve for num_epochs_to_stop:

break // Early stopping

end

end

end

Step 4: Hyperparameter Tuning

hyperparameters = learning_rate: [1e-2, 1e-5], batch_size: [16, 32, 64], num_transformer_blocks: [6, 12] // candidate hyperparameters

best_hyperparameters = grid_search(model, hyperparameters, training_set, test_set) // find best hyperparameters using grid search

Step 5: Evaluation

test_accuracy = evaluate(model, test_set) // calculate test accuracy

metrics = calculate_metrics(model, test_set) // calculate precision, recall and F1 score

### 3.5 Performance metrics

To evaluate the performance of the proposed SkinViT, we considered performance metrics which are as follows:

$$\begin{array}{c} Accuracy=\frac{T_{pos}+T_{neg}}{T_{pos}+T_{neg}+F_{pos}+F_{neg}} \end{array}$$ (10)

$$\begin{array}{c} Recall=\frac{T_{pos}}{\left(T_{pos}+F_{neg}\right)} \end{array}$$ (11)

$$\begin{array}{c} Precision=\frac{T_{pos}}{\left(T_{pos}+F_{pos}\right)} \end{array}$$ (12)

$$\begin{array}{c} F1score=2*(\frac{\left(Precision*Recall\right)}{\left(Precision+Recall\right)}) \end{array}$$ (13)

The *T*_*neg*_ represents the true negative, which means the accurate classification of Nonmelanoma images and *T*_*pos*_ shows the true positive, meaning the accurately classified Melanoma images by the proposed model. False positive *F*_*pos*_ is the wrongly classified Nonmelanoma image as Melanoma while false negative *F*_*neg*_ is the opposite of *F*_*pos*_, meaning misclassification of the Melanoma image as Nonmelanoma. *Recall* measures how often it correctly predicts a result for all samples that should have been classified positive, whereas *Precision* measures how often a method predicts a positive result. The *F*1 − *score* is the harmonic mean of precision and recall, which shows how the classifier predicts correctly.

## 4 Simulation setup and results

This section details the simulation setup and results of the proposed SkinViT.

### 4.1 Simulation setup

The proposed SkinViT model is implemented in the Anaconda environment using Python 3.8 with Tensorflow, Keras, Scikit-Learn, Matplotlib and Numpy libraries installed on Windows OS with system configuration Intel Core i7-11800H @2.3GHz, 16GB DDR4, NVIDIA RTX 3060. The SkinViT model is trained on 3 datasets, as described in the dataset acquisition section with 8473 dermoscopic images in dataset1, 1082 in dataset2 and 9555 in dataset3, as depicted in Table 2. Moreover, we augmented the dataset as mentioned in data augmentation section to avoid overfitting at the same time increase the classifier’s efficiency on unseen images. Furthermore, Adam is employed as an optimizer to update SkinViT parameters in the proposed work during the model training. The epochs and batch size are set to 70 and 16, respectively. The learning rate for the proposed work is set to 1*e* − 5.

### 4.2 Simulation results

This section first discusses various ablation studies related to the proposed SkinViT model. Next, a comprehensive analysis of the performance of the proposed SkinViT is carried out and compared with other SOTA models.

### 4.3 Ablations

The ablations of this work comprise of 1) determining the best value of the L2 kernel; 2) training the model with different optimizers to determine the optimum one; 3) the effect of augmentations and without augmentations on the proposed method; 4) comparative analysis with different kernels classifiers.

1. Tuning the L2 kernel classifier: In this simulation, we experimented with different values of *L*2 to select the best value of *L*2 (Eq 6) for the proposed model. We changed the value of L2 from 0.01 to 1.0 with five intervals in total to select the best result. Table 4 depicts the performance of the proposed SkinViT on different values of *L*2. From Table 5 it can be observed the best result was obtained on 0.1 with 0.9109 on dataset1, 0.8611 on dataset2 and 0.8911 on dataset3.
2. Selection of Optimizer: In this simulation, the proposed model is trained by employing different optimizers to evaluate the classification performance as given in Table 5. The SkinViT performed best with the Adam optimizer achieving the classification accuracy of 0.9109 on Dataset1, 0.8611 on Dataset2 and 0.8911 on Dataset3, which is superior to RMSprop, achieved 0.8996 on Dataset1, 0.8518 on Dataset2 and 0.8733 on Dataset3.
3. Effect of Augmentations on Proposed SkinViT: In this simulation, we evaluated the effect of classification accuracy using the augmentations on training datasets. It can be observed from Table 6 that the proposed SkinViT performed better while using augmentations, this is due to the fact that there was more representation of the training samples for generalization. From the results, it can be seen that augmentations helped the proposed SkinViT to exceed accuracy by 2.72% on Dataset1, 3.24% on Dataset2 and 1.52% on Dataset3.
4. Selection of classifier: In this simulation, we used various classifiers to select the optimal classifier to classify Melanoma and Nonmelanoma and to achieve the best results on the test set. Table 7 details the results of different classifier, it can be observed that the proposed SkinViT achieves the best performance using *L*2 kernel for the classification task which exceed using the Gaussian kernel by 1.72% on Dataset1, 7.4% on Dataset2 and 0.37% on Dataset3. Here it can be observed SkinViT on Dataset2 achieved better accuracy while using the *L*1 kernel than the Gaussian kernel, which is due to the fact that Dataset2 has a small number of training samples, so the more complex Gaussian kernel performed poor while *L*1, which is not complex kernel achieved better accuracy. Overall from Table 8, it can be seen that using the *L*2 kernel has increased the performance of the proposed SkinViT.

**Table 4:** Performance comparison of SkinViT using different values of *L*2.

Dataset	0.01	0.05	0.1	0.5	1.0
Dataset1	0.8961	0.8991	0.9109	0.8943	0.8872
Dataset2	0.8148	0.8148	0.8611	0.8564	0.8379
Dataset3	0.8838	0.8905	0.8911	0.8942	0.8832

**Table 5:** Performance comparison of SkinViT using different optimizers.

Optimizer	Dataset	Melanoma	Nonmelanoma	Overall
Adam	Dataset1	0.9082	0.9139	0.9109
	Dataset2	0.7683	0.9179	0.8611
	Dataset3	0.9087	0.8723	0.8911
RMSprop	Dataset1	0.8894	0.9114	0.8996
	Dataset2	0.8902	0.8283	0.8518
	Dataset3	0.9402	0.8019	0.8733

**Table 6:** Performance comparison of SkinViT using different optimizers.

Dataset	With Augmentation	Without Augmentation
Dataset1	0.9109	0.8837
Dataset2	0.8611	0.8287
Dataset3	0.8911	0.8759

**Table 7:** Comparison of SkinViT performance on different kernels classifiers.

Dataset	SVM (*L*2 Kernel)	SVM (Gaussian Kernel)	*L*1 Kernel
Dataset1	0.9109	0.8937	0.8931
Dataset2	0.8611	0.7870	0.8425
Dataset3	0.8911	0.8874	8780

**Table 8:** Performance Comparison of SkinViT on different datasets.

Dataset	Classes	Accuracy	Recall	precision	F1-score
Dataset1	Melanoma	0.9082	0.9082	0.9235	0.9158
	Nonmelanoma	0.9139	0.9139	0.8969	0.9053
	Overall	0.9109	0.9082	0.9235	0.9158
Dataset2	Melanoma	0.7682	0.7683	0.8514	0.8077
	Nonmelanoma	0.9179	0.9179	0.8662	0.8913
	Overall	0.8611	0.7683	0.8514	0.8077
Dataset3	Melanoma	0.9087	0.9087	0.8836	0.8960
	Nonmelanoma	0.8723	0.8723	0.8996	0.8857
	Overall	0.8911	0.9087	0.8836	0.8960

### 4.4 SkinViT performance analysis

This section describes the performance analysis of the proposed SkinViT on considered datasets. The feasibility of using pretrained model on our custom dataset is dependent upon the nature and characteristics of the dataset on which the model was trained. The lack of unique medical related features means that transfer learning cannot achieve higher level of classification accuracy. Therefore, the proposed SkinViT model is trained from scratch on considered datasets. From Table 8, it can be noticed that SkinViT performed best on Datasset1 with an overall accuracy of 0.9109, whereas it achieved 0.9082 accuracy for Melanoma class and 0.9139 accuracy for the Nonmelanoma class. Dataset1 displayed overall recall of 0.9082, precision of 0.9235 and F1-score of 0.9158. Dataset2 achieved an accuracy of 0.7682 in the Melanoma class and 0.9179 in the Nonmelanoma class, which is the highest accuracy of Nonmelanoma. The overall accuracy of SkinViT on Dataset2 came out to be 0.8611 with an overall recall of 0.7683. Moreover, SkinViT achieved on Dataset2 overall precision of 0.8514 and F1-score of 0.8077. Dataset3 achieved an accuracy of 0.9087 in the Melanoma class, which is slightly higher than Dataset1 and 0.8723 in the Nonmelanoma class with an overall accuracy of 0.8911. Moreover, SkinViT on Dataset3 displayed an overall precision of 0.8836, recall of 0.9087 and F1-score of 0.8960. Dataset1 displayed the highest accuracy, precision and F1-score, while Dataset3 slightly higher recall compared to Dataset1, which is due to higher false negatives because of the minimum similar representation in the training samples of those false classified. Subsequently, Dataset3 displayed the second-best accuracy with the second-best precision and F1-score.

**Fig 3:**
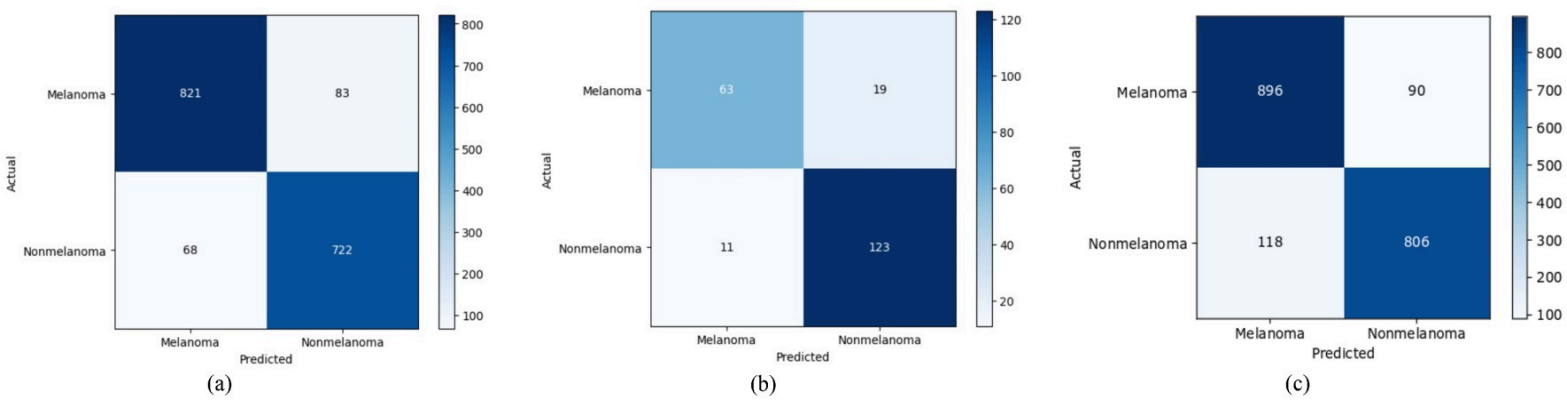
Confusion Matrix of SkinViT on a) Dataset1, b) Dataset2 and c) Dataset3.

Sometimes accuracy alone does not give the complete picture of how well the model is performing. To answer this, the confusion matrix which is graphical representation of the model performance on individual classes, is plotted. From Fig 3, it can be noted that SkinViT on Dataset1 has 821 *T*_*pos*_, 722 *T*_*neg*_, 83 *F*_*neg*_ and 68 *F*_*pos*_. The proposed SkinViT on Dataset2 has 63 *T*_*pos*_, 123 *T*_*neg*_, 19 *F*_*neg*_ and 11 *F*_*pos*_, whereas on Dataset3, SkinViT has 896 *T*_*pos*_, 806 *T*_*neg*_, 90 *F*_*neg*_ and 118 *F*_*pos*_. It can be seen that the proposed SkinViT has the highest accuracy of 0.9109 on Dataset1, followed by 0.8911 on Dataset3 and 0.8611 on Dataset2.

**Fig 4:**
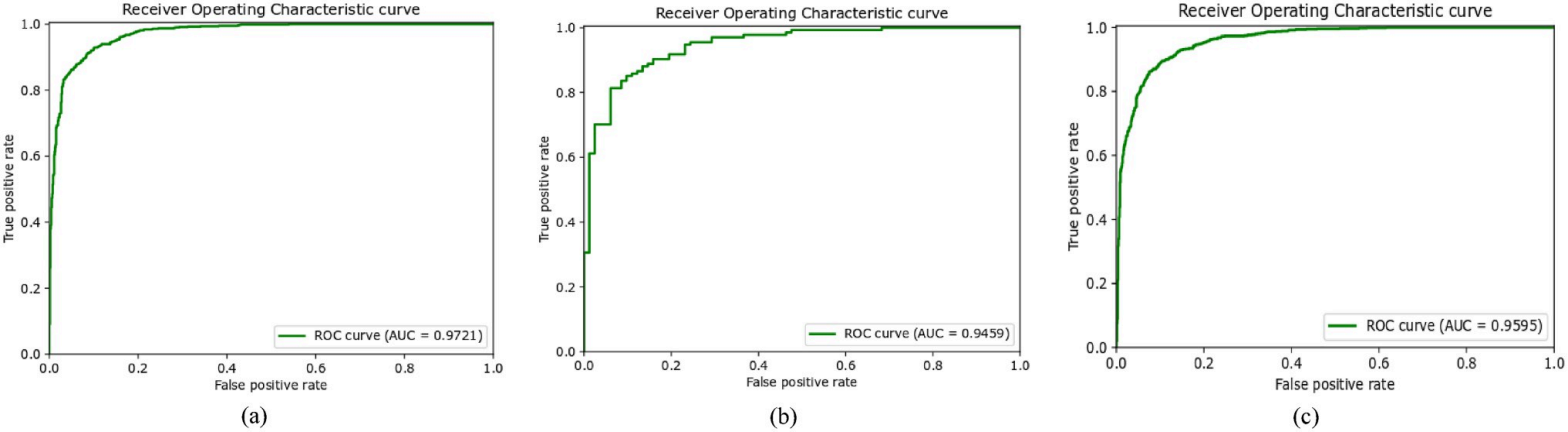
ROC curve of SkinViT on a) Dataset1, b) Dataset2 and c) Dataset3.

**Fig 5:**
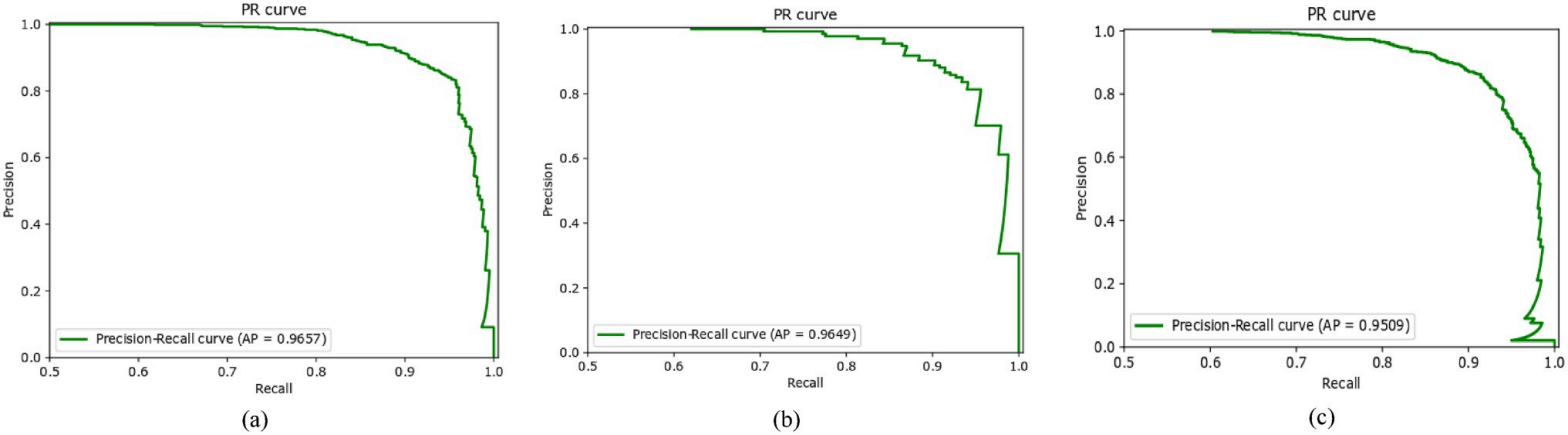
PR curve of SkinViT on a) Dataset1, b) Dataset2 and c) Dataset3.

We have also plotted the Receiver Operating Characteristic (ROC) curve, which tells the model’s ability of predicting over a range of thresholds. The ROC curve is plotted by *TPR* (True Positive Rate) on the y-axis while *FPR* (False Positive Rate) is on the x-axis. The area under the curve (AUC) represents how well the model distinguishes the classes. The AUC of 0.9711 on Dataset1, as illustrated in Fig 4(a), shows that SkinViT has 97.11% chance to accurately classify Melanoma and Nonmelanoma classes. The AUC of 0.9459 on Dataset2, as illustrated in Fig 4(b), means SkinViT has 94.59% chance to accurately classify Melanoma and Nonmelanoma classes. The AUC of 0.9595, as illustrated in Fig 4(c), shows that SkinViT has 95.95% chance to accurately classify Melanoma and Nonmelanoma classes. We also plotted the Precision-Recall (PR) curve, unlike ROC, which considers TN, to examine how well the proposed SkinViT performed while predicting Melanoma images. It can be noted from Fig 5 that the curves are near the top right corner which shows that SkinViT performed well in identifying Melanoma images. SkinViT on Dataset1 achieved 0.9657 AP (Fig 5(a)) while on Dataset2 achieved 0.9649 AP (Fig 5(b)) and on Dataset3 achieved 0.9509 (Fig 5(c)).

### 4.5 Comparative analysis with SOTA models

In this section, we present the performance comparison of the proposed SkinViT with other SOTA models to validate the efficiency of the SkinViT in terms of accuracy metric. We evaluated the performance of different models such as EfficientNetv2 [46], MaxViT [47], MobileViTV2 [48] and ViT [44] on all the considered datasets. It can be seen from Table 9 that the proposed SkinViT performed best on Dataset1 with an accuracy of 0.9109, followed by the second best on ViT 0.8636 which is a gain of 4.73%, recall of 0.9082 which is a gain of 0.77% from ViT with 0.9004, precision of 0.9235 which is a gain of 2.81% from the second best 0.8954 displayed by EfficientNetV2 and the F1-score of 0.9158 which is a gain of 4.01% from the second best 0.8757 by ViT. SkinViT on Dataset2 displayed an accuracy of 0.8611, followed by the second best on ViT 0.8241, which is a gain of 3.7%, recall of 0.7683 which is a gain of 10.98% from the second best 0.6585 displayed by EfficientNetV2, the precision of 0.8514 which is loss of 2.79% from the best 0.8793 achieved by ViT and F1-score of 0.8077 which is gain of 8.17% from the second best 0.7286 achieved by ViT. Similarly, SkinViT on Dataset3 showed the best accuracy of 0.8911, which is a gain of 4.55% from the second best accuracy of 0.8456 displayed by MaxViT, recall of 0.9087 which is a gain of 9.73% from the second best 0.8114 by EfiicientNetV2, the precision of 0.8836 which is a loss of 0.59% from the best 0.8895 by MaxViT and F1-score of 0.8960 which is a gain of 5.35% from the second best of 0.8425 displayed by MaxViT.

**Table 9:** Comparative analaysis of SkinViT and other models.

Dataset	Method	Accuracy	Recall	Precision	F1-score
Dataset1	EfficientNetV2 [46]	0.8412	0.7954	0.8954	0.8424
	MaxViT [47]	0.8418	0.8252	0.8715	0.8477
	MobileViTV2 [48]	0.8141	0.8695	0.7996	0.8331
	ViT [44]	0.8636	0.9004	0.8524	0.8757
	SkinViT (ours)	0.9109	0.9082	0.9235	0.9158
Dataset2	EfficientNetV2 [46]	0.8102	0.6585	0.8060	0.7248
	MaxViT [47]	0.7638	0.5365	0.7719	0.6328
	MobileViTV2 [48]	0.8009	0.6707	0.7746	0.7139
	ViT [44]	0.8241	0.6219	0.8793	0.7286
	SkinViT (ours)	0.8611	0.7683	0.8514	0.8077
Dataset3	EfficientNetV2 [46]	0.8424	0.8114	0.8743	0.8417
	MaxViT [47]	0.8456	0.8002	0.8895	0.8425
	MobileViTV2 [48]	0.8031	0.7809	0.8280	0.8038
	ViT [44]	0.8099	0.7769	0.8427	0.8084
	SkinViT (ours)	0.8911	0.9087	0.8836	0.8960

### 4.6 SkinViT performance on HAM10000

Further, to validate the performance of our proposed SkinViT, we evaluated the performance of the HAM10000 dataset which is used by most of the previously published work. It can be seen from Table 10 that the proposed SkinViT obtained an accuracy of 0.9254, followed by MobileNet by Damian et al. with 0.897 accuracy and STDP based SNN by Zhou et al. with 0.877 accuracy. The higher accuracy of SkinViT is due to the lesser false classifications, which is because of the attention mechanism that helps model learn the desired features more efficiently.

**Table 10:** Comparative analysis of SkinViT with previous work.

Performance Metrics	SkinViT (ours)	MobileNet (Damian et al.) [32]	STDP based SNN (Zhou et al.) [30]
*Accuracy*	0.9254	0.897	0.877

### 4.7 Discussion

In this work, SkinViT displayed the ability of outlooker and self-attention to diagnose Melanoma and Nonmelanoma through dermoscopic images. It can be seen from the results that SkinViT performed better compared to the other CNN and Transformer based models. In contrast to transformers, which can compute the attention of any patch, regardless of its distance, a CNN alone needs to perform additional convolutions to increase the receptive field in order to determine the relationship between any two neighboring pixels, resulting in difficult to possess the ability to perform long-range computation. In SkinViT, outlooker block is used instead of patch embedding component in ViT to learn features whereas self-attention is used to learn important features and ignoring the noisy ones. Results show that the SkinViT performed better compared to CNN and Transformer based models, which validates its superiority over other models.

From the results, it can be noted that the SkinViT performed better on both Melanoma and Nonmelanoma classes. However, CNN model EfficientNetV2 was better in predicting Nonmelanoma images while performed poor in classification of Melanoma as given in Table 9. Moreover, Transformer based method MaxViT and ViT performed better in classifying Melanoma images whereas another hybrid model MobileViTV2 performed well on classifying Nonmelanoma images compared to the Melanoma images. Whereas, SkinViT was equally good in classifying both the classes indicating that SKinViT is robust than using CNN or transformer based models alone in dealing with imbalanced datasets. We also observed from the results that the EfficientNetV2 performed well compared to other Transformer based methods such as MaxViT and ViT.

Most of the researcher used CNN models for Melanoma and Nonmelanoma detection while some used transformer alone architecture for the considered task. To the best of our knowledge this is the first time to use outlooker and transformers for skin cancer detection task with Melanoma and Nonmelanoma (SCC and BCC) classes which are the most frequently diagnosed skin cancer types. Additionally, previous research results were compared with the proposed work as depicted in Table 10. It is essential to point out that each researcher used different classes for their respective problems. Although MobileNet and STDP based SNN showed good accuracy of 0.897 and 0.877 compared to others which took into account Melanoma and Nonmelanoma (Nevus) and our proposed method outperformed both with an accuracy of 0.9254 on the same dataset which validates the efficiency of our proposed method SkinViT. However, our work focused on binary classification problem of Melanoma and Nonmelanoma (BCC and SCC). Moreover, the high accuracy by the proposed method can help early diagnosis of skin cancer and ease burden on dermotologist. This research can benefit researchers to further improve the methodology for the image segmentation to detect abnormalities in dermoscopic images in terms of Melanoma and Nonmelanoma.

Despite the great performance of the proposed SkinViT model compared to SOTA, there are some limitations and challenges in this research. Firstly, the data used for training the proposed SkinViT model from scratch is of moderate size. The size of data significantly impacts the efficacy of training a deep learning model for optimal performance. The greater the amount of data, the higher the efficiency of the model. Therefore, for future research, a large dataset should be curated by combining the publicly available datasets (ISIC archive, HAM10000 etc.) to improve the model efficiency. Furthermore, the dataset used has huge class imbalance which can highly impact the performance of the proposed model. The current research employed the geometric data augmentation technique to handle the class imbalance issue. The future research will explore the use of generative adversarial network (GAN) or advanced data augmentation techniques like MixUp and CutMix which involves combining the multiple images or patches to create new training samples for representation and enhance the model generalization.

## 5 Conclusion

The focus of this research is on the automated detection of skin cancer types, Melanoma and Nonmelanoma (BCC and SCC), which can help reduce the mortality rate by early diagnosis and also help ease the burden on dermatologists. To achieve this goal, we devised a novel deep learning model named SkinViT, which employs transformer blocks, outlooker blocks and MLP Head for classification. Further, the proposed SkinViT eliminates the requirement for high computational power due to its fewer params (around 27.1million), as opposed to other popular classification models such as ViT and MaxViT.

The total number of training samples were enhanced by employing augmentations such as horizontal flip, shear transformation and rotation to resolve class imbalance problem. Moreover, the use of an SVM classifier, specifically the L2 kernel, has increased the optimality of the prediction value by taking the mean of the data to avoid overfitting. We performed multiple simulations to assess SkinViT model performance. It is evident from Tables 9 and 10 that the SkinViT achieved higher classification accuracy in comparison to other methods. This is perhaps due to the outlooker block in SkinViT, unlike ViT, efficiently encodes fine level features by measuring the likeness between token pair representations which is efficient in terms of parameters learning features than convolutions. Moreover, the sliding window adopted in outlook attention locally encodes token representations and preserves important positional information for classification task. Furthermore, the outlook attention weight generation is simple reshaping operation, unlike self-attention which is dependent on query key matrix multiplications. Finally, the proposed MLP head has the SVM L2 kernel classifier that further optimizes the model, which takes the mean of the values for the prediction score. This provides the SkinViT with better feature learning ability which results in higher accuracy in classifying Melanoma and Nonmelanoma in this proposed work.

For the skin cancer detection problem, it is crucial that the false classifications should be minimal to ensure the model’s applicability and reliability in the real-world scenarios. For Melanoma detection, it is imperative to minimize false negative as it may lead to treatment delays and subsequently diminish the 5-year survival rate. While the false positive would only necessitate further diagnostic procedures such as biopsy. The reason for the false classification of Melanoma images as Nonmelanoma (*F*_*neg*_) could be due to insufficient representation of the Melanoma images. As it can be observed from Table 2 that the number of images is Melanoma is significantly lower than that of Nonmelanoma. This can make it difficult for the model to generalize the instances for which it is not trained. Another reason for the false classification can be the excessive noise such as hair and air bubble in the images which make it challenging for the model to learn the important features. The proposed SkinViT method can further be improved in the future using additional datasets available publicly with segmentation task to detect skin diseases. Furthermore, the image quality of the dermoscopic images for Melanoma and Nonmelanoma can further be improved. Also, the classification results of SkinViT can be utilised for implementing a Melanoma and Nonmelanoma recognition system to assist the dermatologists in diagnosis.
